# Surface chemistry and germination improvement of Quinoa seeds subjected to plasma activation

**DOI:** 10.1038/s41598-017-06164-5

**Published:** 2017-07-19

**Authors:** A. Gómez-Ramírez, C. López-Santos, M. Cantos, J. L. García, R. Molina, J. Cotrino, J. P. Espinós, A. R. González-Elipe

**Affiliations:** 1 Departamento de Física Atómica, Molecular y Nuclear, Universidad de Sevilla, Avda. Reina Mercedes, 41012 Sevilla, Spain; 2 Laboratory of Nanotechnology on Surfaces, Instituto de Ciencia de los Materiales de Sevilla (CSIC-Universidad de Sevilla), Avda. Américo Vespucio 49, 41092 Sevilla, Spain; 3 Department of Plant Biotechnology, Instituto de Recursos Naturales y Agrobiología de Sevilla (CSIC), Av. Reina Mercedes, 10, Sevilla, 41012 Spain; 4 Institute of Advanced Chemistry of Catalonia (IQAC), Department of Chemical and Surfactants Technology, Plasma Chemistry Group, Consejo Superior de Investigaciones Cientificas (CSIC), C/Jordi Girona 18-26, 08034 Barcelona, Spain

## Abstract

Plasma treatment is recognized as a suitable technology to improve germination efficiency of numerous seeds. In this work Quinoa seeds have been subjected to air plasma treatments both at atmospheric and low pressure and improvements found in germination rate and percentage of success. Seed water uptake by exposure to water vapor, although slightly greater for plasma treated seeds, did not justify the observed germination improvement. To identify other possible factors contributing to germination, the chemical changes experienced by outer parts of the seed upon plasma exposure have been investigated by X-ray photoemission spectroscopy (XPS) and scanning electron microscopy (SEM-EDX). XPS revealed that the outer layers of the Quinoa plasma treated seeds were highly oxidized and appeared enriched in potassium ions and adsorbed nitrate species. Simultaneously, SEM-EDX showed that the enrichment in potassium and other mineral elements extended to the seed pericarp and closer zones. The disappearance from the surface of both potassium ions and nitrate species upon exposure of the plasma treated seeds to water vapor is proposed as a factor favoring germination. The use of XPS to study chemical changes at seed surfaces induced by plasma treatments is deemed very important to unravel the mechanisms contributing to germination improvement.

## Introduction

Coming from the Andes, Quinoa (Chenopodium quinoa, Willd) is a native food crop tolerant to high stress conditions such as high altitudes (up to 4000 m.o.s.l.), drought, extreme temperatures, high salinity (40 mS cm^−1^) and sandy soil with very low levels of nutrients. Due to its high nutritional value, associated with a high content of vitamins (B1, B9, C and E) and healthy lipids, Quinoa is deemed a key plant against famine in regions affected by crop failure. Moreover, very likely because its excellent protein amino acid balance very adequate for celiac individuals, Quinoa has become a fashionable food in developed countries where it has reached prices (around 10 euros per kilogram) surpassing that of other cereals. All these features have contributed to gradually spread the Quinoa farming all over the world, even though the development of suitable agricultural practices according to market trends and climate change is still a challenge.

Quinoa seeds are spherical with a mean diameter of 1.4–1.6 mm^1^. Their outer parts are formed by a two-layered pericarp and a seed coat, which is bitegmic (constituted by a testa and a tegmen) and has a thickness varying between 34 µm for wild forms and 15.85 µm for cultivated ones. Inside, a peripherical curved embryo surrounds a large central perisperm made of starch and covered by integuments. A micropylar endosperm in the form of a cone surrounds the radicle tip. This embryo consists of a characteristic hypocotyl-radicle axis with two cotyledons. Protein and lipid bodies are located in the embryo and endosperm.

Seed germination is a process where a multitude of biochemical changes occur. Germination starts by water uptake, followed by radicular emergency that, for the quinoa in Andean regions, occurs 6–10 h after imbibition when the seed water content increases from 41 to 45%^2^. In the seeds of some species, coat controls germination by acting as a physical barrier that prevents radicle protrusion^3, 4^ and imposing a mechanical restraint that interferes in the water imbibition. Thus, environmental conditions may greatly influence seed release rate with high temperatures and long photoperiods favoring the occurrence of dormant seeds^5^. These conditions are common in Europe, particularly in the South, where they will become more frequent in parallel to the progression of climate change. This will provoke that, in the future, a smaller number of quinoa varieties may be successfully cultured in these regions.

During the last years, different techniques using plasmas have been proposed to improve the germination efficiency of seeds and subsequent plant growth. For example, Park *et al*.^6^ proposed the plasma treatment of the water used to irrigate the seeds as a means to enhance plant growth and to reduce considerably the amount of required water. Similarly, the direct exposure of the seeds to non-thermal plasmas is a relatively modern practice that has been demonstrated rather efficient to accelerate or improve germination of a large number of plants^7–11^. However, despite the numerous evidences of germination and plant growth improvement available in literature, a general explanation to account for these beneficial effects of plasma is not yet available. Besides, finding a general explanation for plasma induced germination faces an additional problem in the sense that the term “non-thermal plasmas” encompasses a large variety of plasma procedures with quite significant differences in operational parameters, working pressure, concentration of active species, electron density, magnitude of electrical field, etc. In general, two main factors have been claimed to account for the generally observed improvement of germination (although this result is by no means universal^12–15^): i) increase in the water uptake through a partially etched seed coat^11, 16, 17^ and ii) the sterilization of the plasma treated seeds^18–23^. Other evidences such as the conversion of the surface state of the plasma treated seeds in hydrophilic^10, 24–26^ or the occurrence of coat plasma etching observed by electron microscopy analysis^10, 17, 25, 27^ have been also encountered.

In the present work, in addition to classical methodological approaches utilized in the literature to investigate the effect of plasmas on seeds, we propose the use of the X-ray photoemission spectroscopy (XPS) to study the surface chemistry of the Quinoa seeds exposed to plasma and then to water vapor to promote their germination. The obtained results evidence that the surface chemistry of these seeds experience great transformations that might be related with the observed increase in germination capacity (for this analysis a seed has been considered as germinated when root and cotyledons emerged). These results have been complemented with other by Scanning Electron Microscopy and Energy Dispersive X-ray Spectroscopy (SEM-EDX) that provide both laterally resolved chemical information and the capacity to investigate inner layers of the seed (i.e., embryo, radicle and perisperm). For this study we have used two types of air plasmas: an atmospheric pressure dielectric barrier discharge (DBD) plasma^28^ and a low pressure radiofrequency^29^ (RF) plasma. Although differing in treatment time, these two plasma treatments produced an improvement in the germination capacity of the Quinoa seeds and induced similar transformations of surface chemistry after plasma and then water vapor exposures. As a result of these investigations, the segregation of potassium and other mineral elements to the outer zones of the seed and the formation of surface nitrate species during DBD plasma treatment are proposed as possible factors contributing to enhance the germination of the Quinoa seeds.

## Results

### Germination and plasma treatments

**Figure 1:**
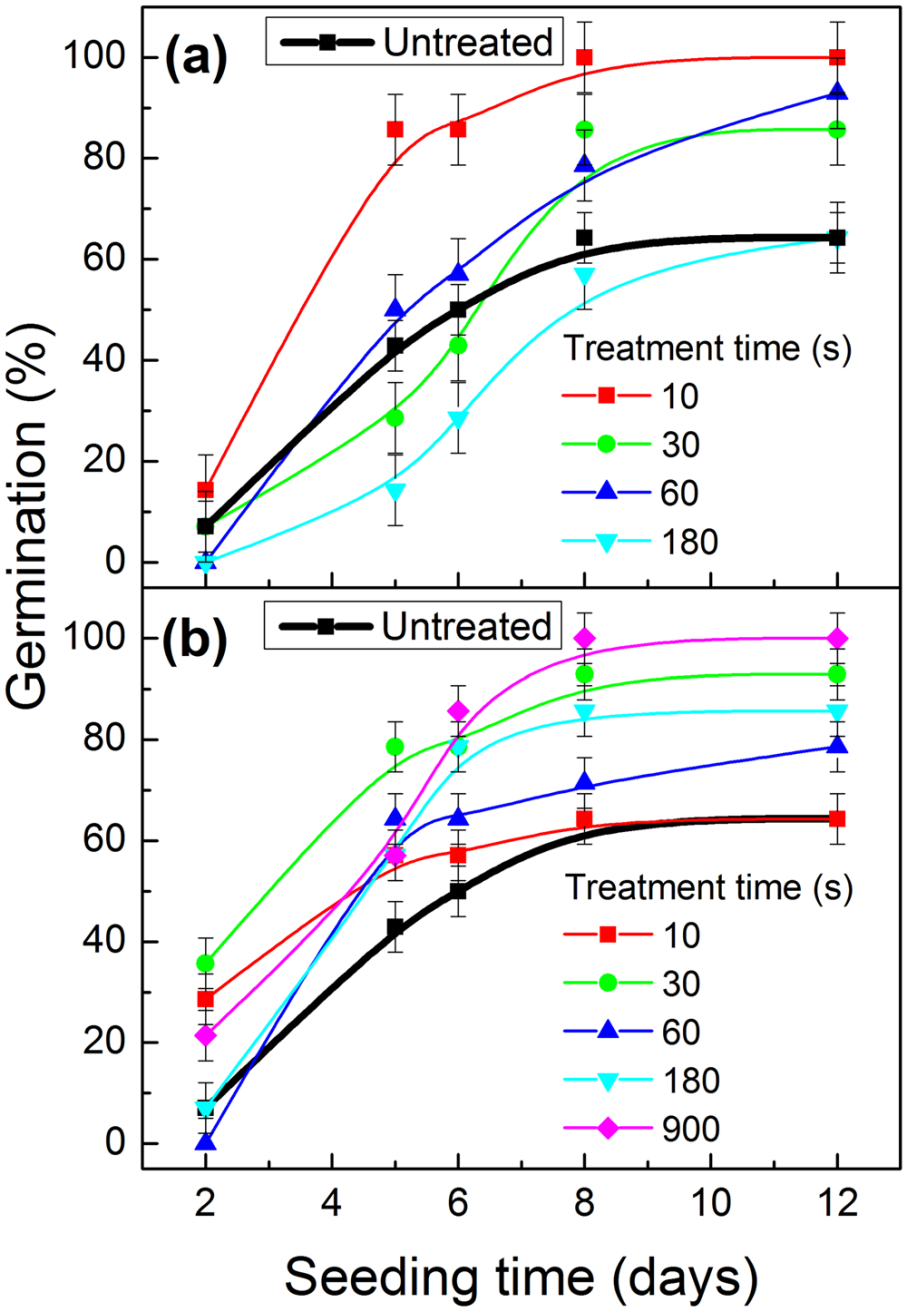
Plots of germination percentages as a function of seeding time for quinoa seeds plasma treated for the indicated periods of time in the RF (top) and DBD (bottom) reactors.

A general result of the plasma activation experiments was a germination improvement for the plasma treated seeds with some differences depending on the type of plasma and treatment time. A compilation of percentages of germinated Quinoa seeds as a function of seeding time for increasing periods of plasma treatment times is gathered in Fig. 1. Figure 1a shows that, in comparison with untreated seeds, those treated with RF plasmas for 10 s experienced an improvement in germination, reaching almost 100% after five days. Plasma treatment times of 60 s produced a germination success rate of 80% after 8 or more days. However, a considerable decrease in germination percentage was obtained for seeds treated for 180 s, indicating that, as found by others, an excess of RF plasma might be deleterious for seed germination (see in supplementary information, Figure S1, the surface damage induced on seeds that were RF treated for long periods of time)^14, 15, 20^.

Unlike the short treatment times required with RF low pressure plasmas, much longer treatments were required when using DBD plasmas to enhance the germination rate of Quinoa seeds. The compilation of results reported in Fig. 1b clearly reveals that treatments for 10 s are rather inefficient to increase germination and that treatment times as high as 900 s are required to achieve 100% germination 8 days after sowing. For intermediate plasma treatment times, the germination rates found ranged between those of the untreated seeds and the longest treatment times.

### Water uptake

**Figure 2:**
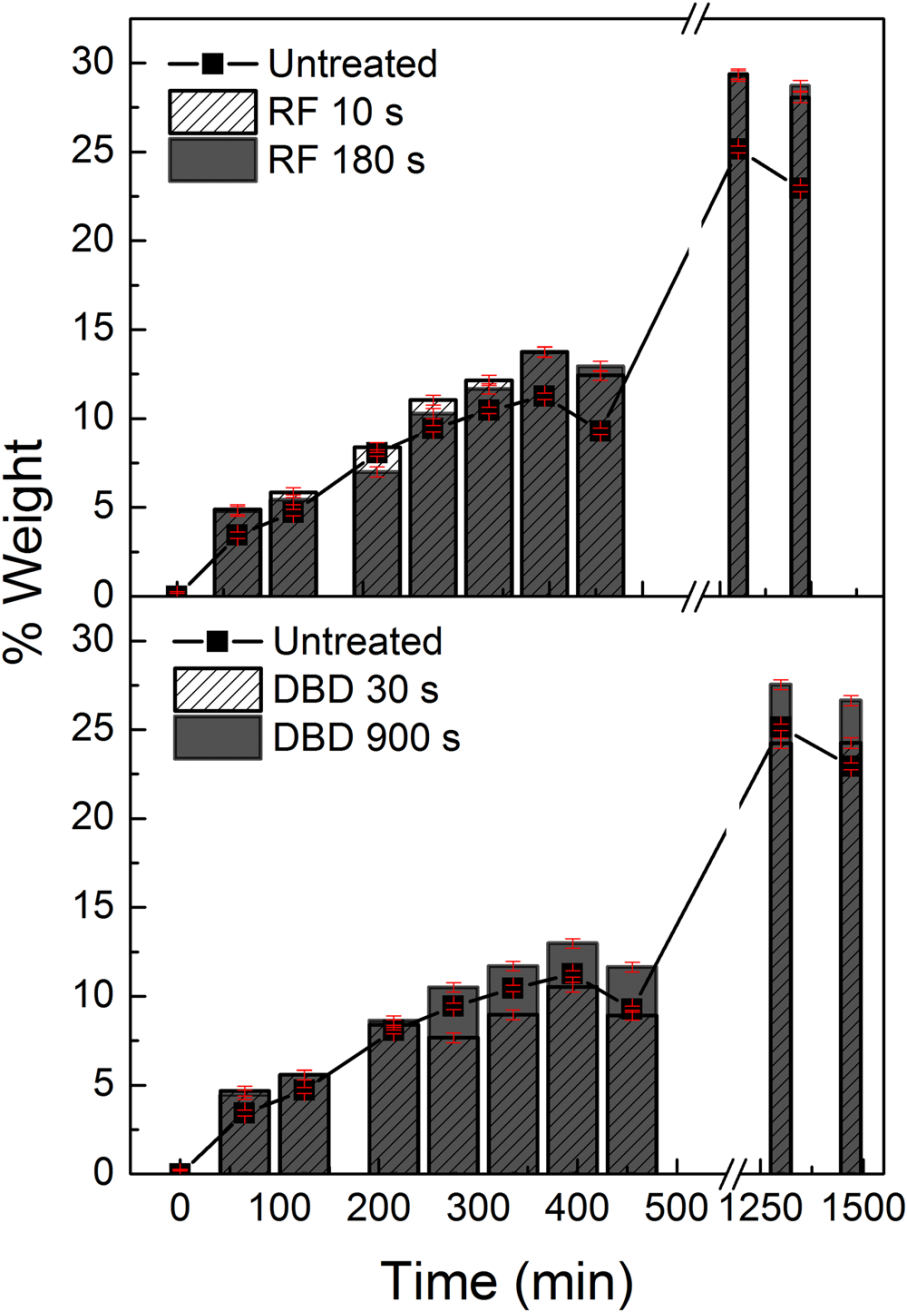
Relative variation of weight for the untreated and plasma treated seeds as a function of exposure time to water vapor for the indicated periods of time. Top) RF and bottom) DBD plasmas.

One of the factors proposed in literature to explain the increase in germination rates usually found for plasma treated seeds is an enhancement in water uptake when the seeds are exposed to water vapor or immersed in liquid water^10, 26^. A first control experiment with untreated seeds immersed in liquid water showed that the weight increase during the first 48 hours followed a sigmoid curve (see supplementary information Figure S2). This evolution suggested a period of rapid uptake between 0 and 5 hours followed by a slower uptake that reached saturation after 48 hours with a maximum water uptake of 165%. A more detailed analysis of the water uptake by the untreated and plasma treated seeds was carried out by exposure to saturated water vapor at room temperature. The results of this series of experiments are reported in Fig. 2. The bar diagrams in this figure reveals that the treated plasma seeds increase their weight by only a slightly higher amount in comparison with the untreated plasma seeds (square dots). Although this result indicates an easier hydration of the plasma treated seeds, the little differences found do not seem sufficient proof to link the increase in germination rate reported in Fig. 1 with an enhancement of water uptake after plasma treatment.

### XPS analysis of the surface chemistry of seeds

XPS analysis of the plasma treated seeds revealed very important modifications in the surface chemistry of the pericarp. This surface sensitive technique provides information about the chemical state of the elements present in the outer layers of the investigated samples^30^ (average depth of analysis of approximately 2 nm). Surface composition in atom percents of the XPS analyzed seeds before and after DBD and RF treatments for various periods of time and for the treated seeds exposed to water vapor are reported in Table 1. In this table, the atomic percentages of the untreated seeds reveal that the surface is rich in carbon, has a certain amount of oxygen and much smaller percentages of nitrogen and potassium. These percentages are similar to those expected for the outer coat of this kind of seeds^31^. The variations in atomic percentages reported in Table 1 prove that the surface chemistry of the pericarp is much affected by both the plasma treatment and the posterior exposure to vapor pressure. Exposure of the original seeds to water vapor for 24 h did not induce any significant modification in surface composition except for a small increase in nitrogen. Exposure of the seeds to the plasma for a short period of time (i.e., 30 s DBD or 10 s RF) induced a significant increase in the content of oxygen and nitrogen in detriment of carbon and a small increase in the content of potassium (c.f., Table 1). A similar increase in the oxygen and nitrogen contents was monitored by SIMS for various seeds exposed to RF air plasmas^26^. For longer plasma exposure times (i.e. 900 s DBD or 60 s RF) changes were even more drastic with additional increases in the percentages of oxygen, nitrogen and potassium. A first assessment of these changes in surface composition suggests that the air plasma produced an important oxidation of the outermost layers of the seeds as evidenced by the increase in the surface oxygen content after the treatment. This is an expected effect due to the highly oxidative character of the excited species in air plasmas^32^ (i.e., atomic oxygen (O^*^) as reported by Kuzminova *et al*.^33^ for this kind of plasmas, and very likely other charged species, such as O_2_^+^) and the ozone simultaneously formed during DBD plasma treatments^34^. Similar surface oxidation effects have been described for different seed grains exposed to RF air plasmas^26^ and for polymers^30^ graphites^35^ and other carbon reach materials^36^ where affectation thicknesses up to 100 nm have been induced by similar plasma treatments^30^. Unlike the negligible effect of water uptake on the surface composition of untreated seeds, plasma treated seeds underwent additional changes upon water vapor exposure. According to Table 1, some potassium disappeared from the surface and the atomic percentage of oxygen varied differently depending on plasma treatment. We tentatively assume that these changes could be related with the changes found in germination efficiency (Fig. 1).

**Table 1:** Elemental surface composition in atom percentages for the Quinoa seeds subjected to different plasma and water exposure treatments.

Seed treatments	%C	%O	%N	%K
Untreated	88.87	9.47	0.72	0.26
Untreated + water vapour (24 h)	87.80	10.73	0.96	0.25
DBD 30 s	73.30	24.89	0.81	0.85
DBD 900 s	57.16	35.96	3.12	3.58
DBD 900 s + water vapour (24 h)	70.94	25.34	3.07	0.49
RF 10 s	79.26	16.59	2.36	1.79
RF 10 s + water vapour (24 h)	63.95	29.53	5.25	1.27
RF 60 s	71.34	20.01	2.62	6.03
RF 60 s + water vapour (24 h)	67.40	25.46	4.74	2.39

**Figure 3:**
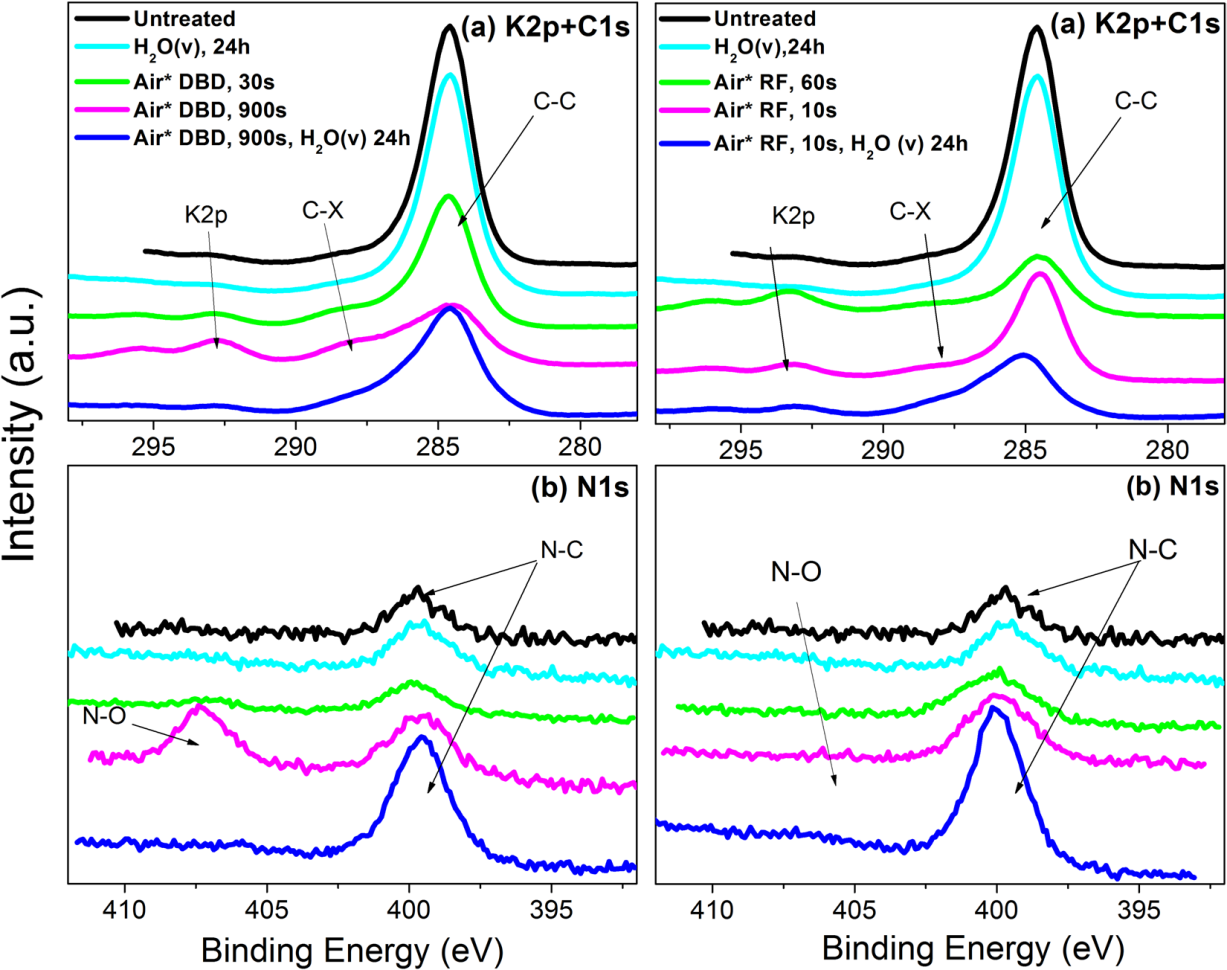
Carbon C1s (**a**) and nitrogen N1s (**b**) high-resolved XPS spectra of Quinoa seeds DBD (left) and RF (right) plasma treated for different treatment times. Curves corresponding to the untreated seeds, the 900 s DBD and the 10 s RF treated seeds after water exposure (24 h) are also shown.

In addition to the atomic surface compositions gathered in Table 1, XPS provided information about the chemical state of the detected elements. Figure 3 shows the series of C1s (including the K2p signal) and N1s XPS spectra that were recorded after various DBD and RF treatments. O1s spectrum was also recorded but its shape and the peaks binding energies (530 eV) was little affected in the curse of these experiments and will not be further discussed here (see supplementary information Figure S3). The C1s/K2p and N1s spectral regions reported in Fig. 3 provide additional information on the chemical changes affecting the seed surface coats when subjected to the plasma/water vapor experiments. The C1s spectrum of the original sample was characterized by a very intense signal at 284.6 eV attributed to C-C and C-H bonds typical of an organic membrane^37^. A small contribution at around 288 eV revealed the presence of C-O (and C-N) functional groups in the analyzed surface layers as expected for a pericarp formed by proteins and other organic macromolecules. According to the C1s spectra of the plasma treated seeds, exposure to plasma produced a progressive increase in the region around 288 eV which we attributed to an increase in the surface concentration of C-OH and C-O-C functional groups^38^, and a significant decrease in the C-C/C-H contribution. This evolution proves the progressive plasma/ozone oxidation etching of the outer layers of the coat, already evidenced by the increase of oxygen atomic percentage reported in Table 1. Similar effects in the C1s peak shape are common for organic materials exposed to oxidant plasmas where the said treatments have been claimed to induce surface hydrophilicity both on organic materials and seed surfaces^26, 38^. In addition to this extensive surface oxidation, the C1s/K2p spectra confirm the already mentioned increase in potassium. Tentatively, we assume that the diffusion of this cation from the interior of the seed to its outer layers might be driven by the polar character of the oxidized functional groups formed at the surface upon plasma exposure and/or to changes in surface potential during plasma activation^39^. In parallel to these changes, the N1s spectra provide other clues on surface chemistry that might be related with the observed improvement in germination. In the spectra reported in Fig. 3b and c there is a common signal at 399 eV that can be attributed to N-H or N-C functionalities present in the macromolecules of a biological membrane^37^. In addition, another rather intense signal at 406 eV appeared in the spectrum of the seeds treated with the DBD plasma for 900 s (the intensity of this signal depended on treatment time and had very little intensity for RF plasmas). This peak, attributed to the formation of NO_3_^−^ and/or NO_2_^−^ functionalities^40^, disappeared after water vapor exposure, while the component at 399 eV significantly increased. We attribute the formation of NO_3_^−^/NO_2_^−^ groups after air plasma treatment to either the oxidation of the N-H, N-C groups in the organic chains of the coat and/or to the adsorption of NO_x_ molecules formed in the air plasma by combination of excited oxygen and nitrogen species^32^. This latter possibility would be supported by previous results with DBD air plasmas working in ozone mode^41^ (see method section) showing the production of a certain amount of NO_x_, although in our experiment the amount should be very little or negligible because we were unable to detect either NO^*^_x_ species in the plasma by optical emission spectroscopy or stable NO_x_ molecules by gas analysis (see experimental section and supplementary information S4). Whatever its origin, the surface removal of these nitrogen oxidized species upon water exposure suggests their diffusion into the interior of the seeds accompanying the potassium ions that became also removed by this water exposure treatment.

### SEM-EDX analysis of the surface morphology and chemistry of plasma treated seeds

Oxidation by air plasma and/or ozone of organic membranes not only produces chemical changes in their outer surface layers but also induces a progressive etching that should manifest by changes in surface topography^26^. Trying to get additional information about the surface morphological changes of pericarp after plasma treatment, we have carried out a thorough analysis by SEM-EDX according to the procedure described in the experimental section. EDX analysis of transversal and longitudinal sections of the seeds has also provided information about the in-depth distribution of minerals and other essential elements within the seed and the existence of possible correlations with the chemical changes observed by XPS at the very external surface layers.

**Figure 4:**
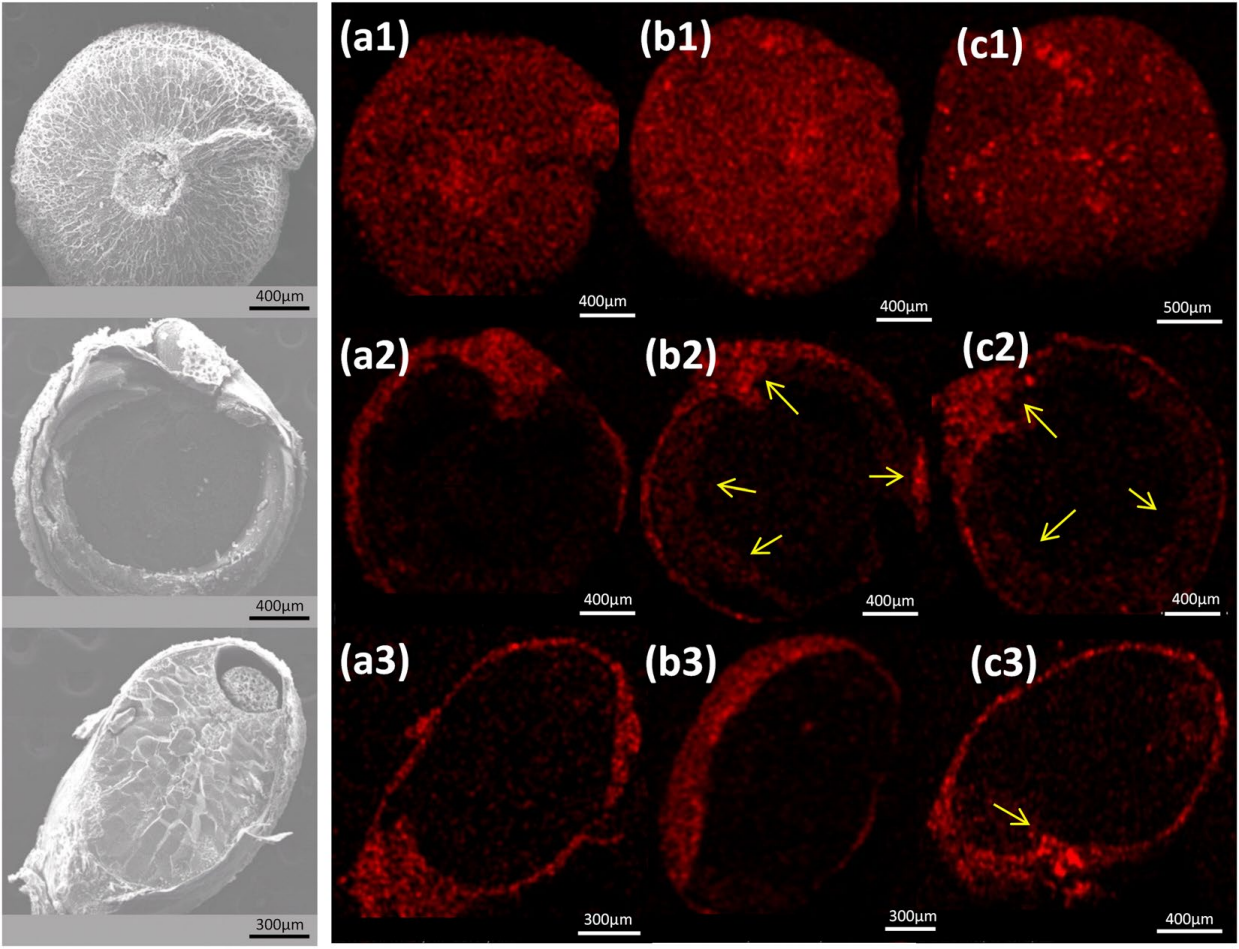
Left) Low magnification SEM micrographs for a Quinoa seed in its original form (top) and for two sections along transversal (center) and longitudinal (bottom) planes. These images clearly show the perisperm, the embryo, the radicle tip, the seed coat and the pericarp (see refs 1 and 42). Right) EDX maps of potassium for equivalent observation zones of original seeds “as received” (a1) and after 15 min (b1) and 50 min (c2) DBD plasma treatment. Similar maps for transversal (a2–c2) and longitudinal (a3–c3) sections of plasma treated seeds. The yellow arrows in the images indicate K migration to these parts.

Left panels in Fig. 4 show a series of low magnification SEM images corresponding to a Quinoa seed and two sections along longitudinal and transversal plans. At this low magnification no significant differences could be appreciated in surface morphology before or after plasma treatments. However, the EDX potassium maps showed variation in the distribution of this element as evidenced Fig. 4 by changes in the intensity and homogeneity of color distribution. For the original seeds, the comparison between maps a1, b1 and c1 clearly show an increase in color intensity for b1 map (i.e. after 15 min DBD plasma treatment) and a more heterogeneous color distribution in c1 (i.e., after 50 min treatment). It is interesting that these changes could be partially reverted by exposure of the plasma treated seeds to water vapor (see supplementary information Figure S5). In line with the XPS results in Fig. 3, these changes support that plasma treatment promoted the enrichment of potassium at the external layers of the seed (note that the EDX average observation depth is around 1 micron, while for XPS is only 2 nm). The maps taken for sections of the seeds before and after plasma treatments (i.e. a2–c2 and a3–c3) confirm this tendency, as well as a preferential enrichment in potassium at the embryo and the radicle tip (see yellow arrows in Fig. 4).

**Figure 5:**
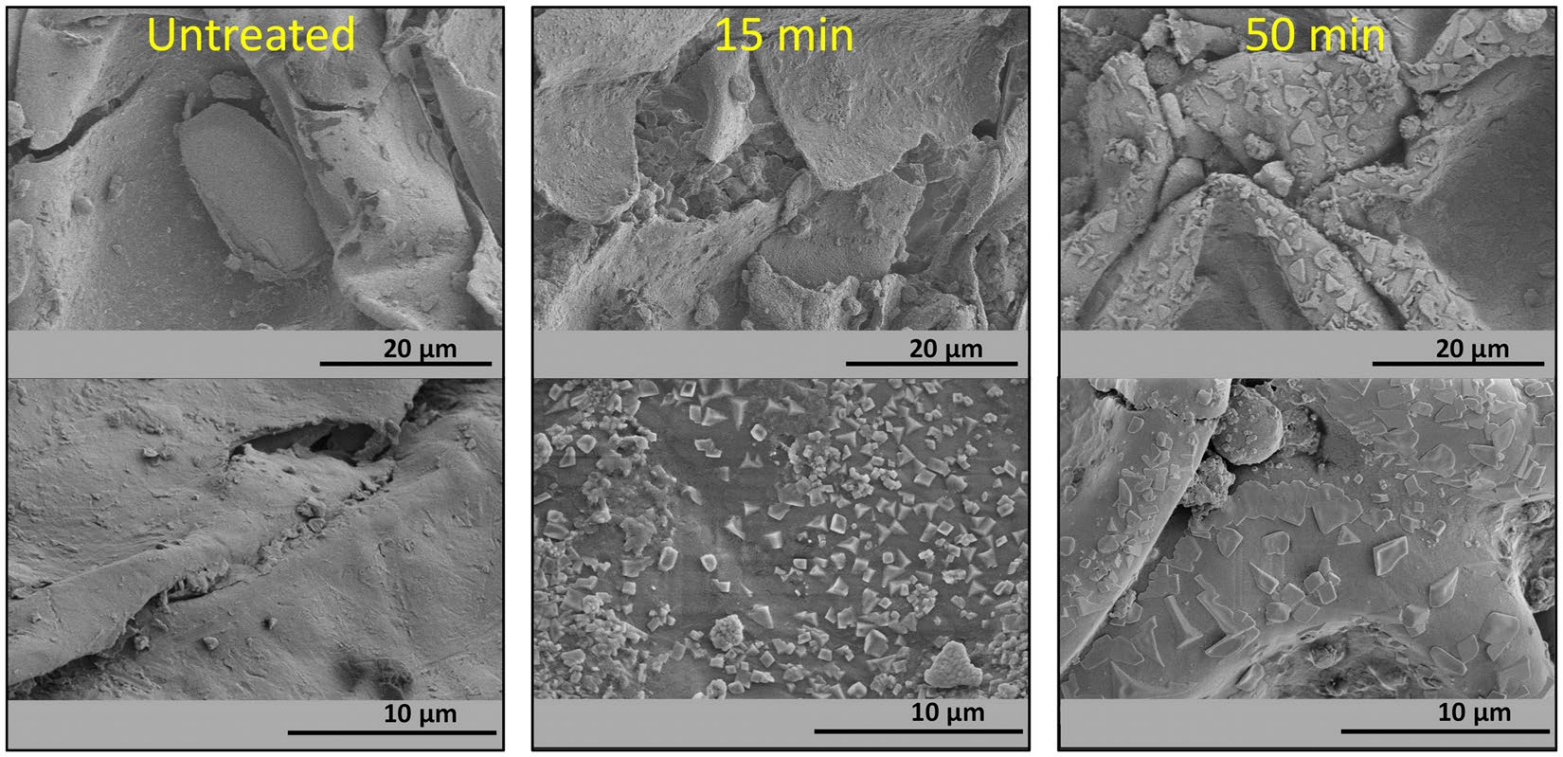
High magnification SEM images (top to bottom) of Quinoa seeds as received (left) and subjected to 15 (middle) and 50 min (right) DBD treatments.

In a similar way that for potassium, the distributions of other minority elements present in the Quinoa seeds was monitored by SEM-EDX analysis. Chemical maps of P, Ca, S, Cl and Mg taken for sections of seeds in their original form and after plasma treatments for increasing periods of time (see supplementary information Figure S6) revealed changes in the distribution of these elements that, particularly for P and Mg, followed a similar tendency than that found for potassium, i.e., an increase in their concentration in the embryo and the outer regions of the seed. All these results prove that plasma treatment of Quinoa seeds, besides affecting the pericarp through plasma etching (see morphological changes in Fig. 5 and supplementary information Figure S1 ^26^), also induces the inner diffusion of mineral and essential elements favouring their accumulation in the outer zones of the seeds (the fact that only K was detected by XPS at the outermost surface layers must be attributed to a higher mobility for the cations of this element, their different sensitivity for XPS detection and/or their different concentration within the seed).

Higher magnification analysis by SEM revealed significant changes in surface morphology after DBD plasma treatment. The SEM images in Fig. 5 show that 15 min DBD plasma treatments induced the formation of surface agglomerates with well-defined geometrical shapes that, after 50 min plasma treatment, evolved into larger and flatter protrusions. The chemical nature of these agglomerates is still unknown because no specific compositional features associated to them could be detected by SEM-EDX analysis. We tentatively propose that the surface segregation and/or condensation of pericarp components, a process that would be promoted by the plasma etching of pericarp, form these agglomerates.

It is noteworthy that after RF plasma treatments agglomerates with not well defined shapes were also detected on the seed surface and that, at given sites, the pericarp was removed or damaged (see supplementary information S1).

## Discussion

Water uptake, together with respiration, is one of the most common methods for monitoring germination^43, 44^. According to Sigstad and Prado^45^ water uptake is a first step for germination, a process that concludes with radicle emergence. Qualitatively, the time evolution of water uptake found here for Quinoa (see Fig. 2 and Supplementary information Figure S2) follows a similar trend than in the majority of seed germination works. Therefore, in line with these previous studies, we attribute the first period of rapid uptake to a simple wetting of the seed tissues, and the slower final uptake to metabolic causes. Between these two stages no lag phase seems to exist for the case of the rapidly germinated Quinoa seeds (e.g., after 48 hours a 7.1% of seeds had already germinated, see Fig. 1). In Chenopodium species in general, and particularly in Quinoa, dormancy is usually attributed to a coat effect that, as demonstrated by Ceccato *et al*.^46^, can be avoided by perforation. These authors also proposed a relation between thickness of the seed coat and dormancy break in different Quinoa accessions.

**Figure 6:**
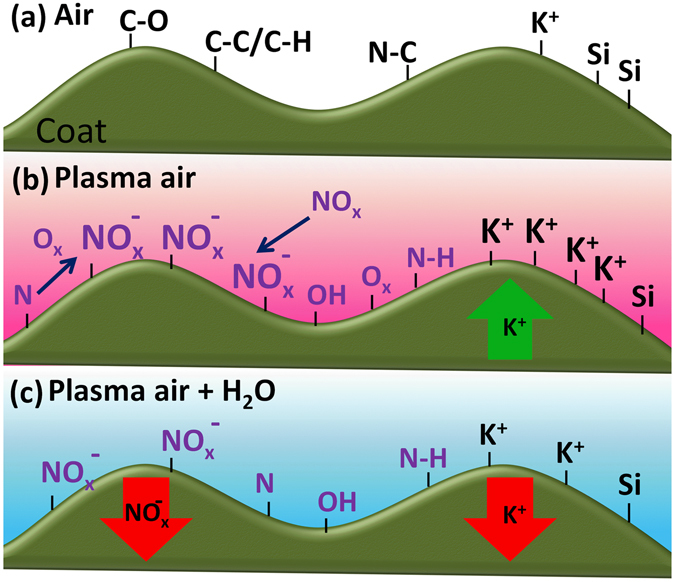
Schematic diagram of the chemistry of the seed surface of original seeds (**a**), after treatment with DBD air plasmas (**b**) and posterior exposure to water vapor.

In the present work we have shown that plasma treatments positively influence germination and extensively affect the external parts of seeds. Our XPS and SEM-EDX results have shown the occurrence of chemical and morphological effects, likely resulting from the etching and damage of the pericarp surface. It seems likely that the observed improvements in germination are linked with the plasma etching of the coat surface (see for example the work of Bellmanm *et al*.^8^) and/or to other biochemical processes triggered by this etching and resulting in the formation of new functional groups at the outermost coat layers^10^, and the enrichment of minority elements at these external zones. These series of effects would favor water uptake and/or induce the seed diffusion of chemicals beneficial for the germination process. Recent studies (Seashore Paspalum, Andean blueberry and Cinchona pubescens) have shown that soaking different seeds into a nitrate water solution favors germination^47–49^. The effect is similar when seeds are soaked in plasma treated water, a procedure well-known to generate nitrate, peroxide and other chemicals in the solution^50^. The XPS and SEM-EDX results reported here have shown that the chemistry of the outer coat layers is drastically affected by the plasma and that long plasma treatments promoted the incorporation of oxidized functional groups that, as reported in numerous studies on seed plasma treatments^10, 24, 25^, must transform the surface state in hydrophilic. In addition, without discarding other possible factors, we propose that the observed surface enrichment in potassium and nitrogen functional groups, particularly NO_x_^−^ groups, clearly observed after DBD plasma treatments might play a role in the enhancement of germination. Figure 6 shows a schematic of the chemical processes taken place on the seed surface after DBD plasma and water exposure treatments (note that etching and segregation effects observed by SEM are not taken into account here). According to this scheme potassium and NO_3_^−^/NO_2_^−^ species once diffused/formed to/at the surface are removed after water exposure, likely by diffusion to the interior of the seed through the partially etched and oxidized outer layers of the coat membrane. We proposed that diffusion of nitrate functional groups to the interior of the seed may contribute to improve germination in a similar way that for seeds soaked in nitrate rich water. It is likely that plasma treatments enhance this diffusion ability by disrupting the chemical bonds at the large macromolecules forming the outer layers of the seed coat.

A significant difference between DBD and RF plasma regarding germination is the need of increasing plasma exposure time when using atmospheric pressure plasmas. A higher ion concentration and energy in RF, a different concentration of other active species in the plasma and their much shorter mean free path at atmospheric pressure plasmas may account for this difference^32^. In addition, regarding the chemical state after RF plasma, XPS has shown that seeds surface state is similar as far as potassium and carbon are concerned, but differ in the much lower amount of NO_x_^−^ species formed at the surface after RF plasma treatment, indicating that the contribution of processes as those schematized in Fig. 6 should be much lower or negligible in this case. Clearly, additional studies addressing the specificities of these two types of plasma in terms of chemical reactivity^24^, possible effect of electric fields^51, 52^, ion bombardment effects or the evolution and fate of the newly generated chemical species at the surface should be further investigated.

## Conclusions

To account for possible factors favoring the germination of plasma treated Quinoa seeds, in this work we have applied the surface sensitive XPS technique to determine the surface chemical changes induced by exposure to air plasmas (DBD and RF) for increasing periods of time. The improvements in germination found after these treatments have been related with the occurrence of specific surface chemical transformations due to the plasma treatments and the posterior modifications induced by the exposure to water vapor. Oxidation of the outer layers of the seed coat and pericarp, formation of oxidized nitrogen species and diffusion of potassium (and other mineral elements to the seed coat) are some of the most significant effects induced by plasma treatment. We have concluded that diffusion phenomena for- and back-wards the seed core are favored due to the polar character of the partially oxidized and etched outer surface layers. In particular, we have proposed as a hypothesis that migration of potassium and, when formed, nitrate species to the interior of the seeds upon water vapor exposure contributes to improve the germination of Quinoa. Beyond these particular inferences from the present experiments, another general conclusion of this work is that applying chemical speciation techniques sensitive to the plasma affected outer zones of the seed coat and/or pericarp is essential to unravel the factors contributing to enhance germination of seeds subjected to plasma treatments.

## Methods

### Quinoa seeds

Mature seeds were harvested from plants of Chenopodium quinoa, Willd. var. atlas growing in the Vitrosur S.L.U. fields. Seeds were taken in their commercial format and used without any additional treatment. This variety was developed by Wageningen University in the Netherlands. ‘Atlas’ is adapted to Europe’s long days and climate conditions (early maturing with a growth cycle of 6 months) and is characterized by a very low saponin content. Plants of this variety are tall (1–1.8 m. according sowing dates), leafy, characterized by late flowering and very late ripening. It is mildew resistant and color of grain is white cream and has a medium size.

### Plasma treatments

**Figure 7:**
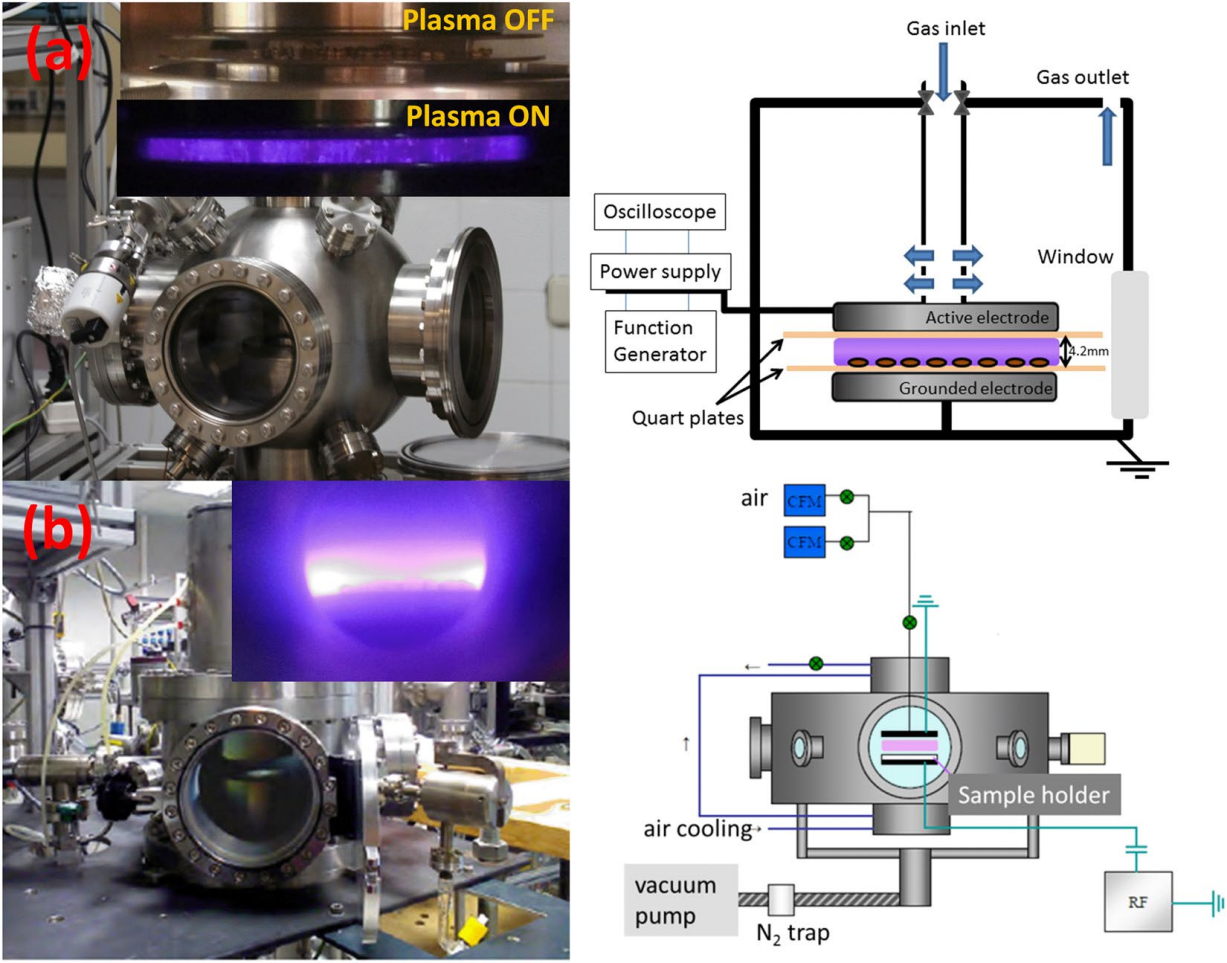
DBD (**a**) and RF (**b**) reactor set-ups and electrical and operational schemes in each case.

Quinoa seeds were treated for progressive times in a dielectric barrier discharge (DBD) and a radiofrequency (RF) plasma reactors operated with dry air at a pressure close to atmospheric conditions (500 mbar) and low pressure (0.1 mbar), respectively. Both discharges were operated in a continuous wave mode. The DBD reactor consisted of two parallel-plate circular metal electrodes covered by quartz plates acting as dielectric barriers. Diameter of metal electrodes was 8 cm, while dielectric disc one was 10 cm, avoiding arching formation due to border effects. Inter-electrode distance was fixed at 4.2 mm, and seeds were situated on the gounded electrode (bottom one), as shown in Fig. 7a. The active electrode was connected to a high voltage power supply (Trek, Model PD05034) and a function generator (Stanford Research System, Model DS345). V(t) and I(t) signals were recorded by means of an oscilloscope (Tektronix TDS2001C- bandwidth 50 MHz, sample rate on each channel 500 Ms/s), using a high voltage probe (attenuation factor: 1:1000) and a current probe (coil with a conversion factor of 0.05 V mA^−1^), respectively. The frequency and the voltage amplitud of the input signal was mantained constant at 1 kHz and 8.2 kV, respectively. Under these conditions the current though the plasma reached a value of 7 mA. Consumed power, in the order of 6.4 W, was calculated from the area of the Lissajous plot Q(t)-V(t), where Q(t) was obtained directly by integrating I(t)^53, 54^. I(t), V(t) curves and the Lissajous plot are shown in the supplementary information (Figure S7). Although those curves do not provide clear hints of filamentary discharge, we observed a higher intensity of the glow light in turn of the individual seeds. We attribute this localized enhancement of light emission to the dielectric character of the seeds. Measurements were performed under static gas flow conditions (i.e, in a close environment during the discharge). Furthermore, working slightly below atmospheric pressure (500 mbar) enabled a smooth ignition of the plasma and avoided typical air microdischarges. Seeds, distributed evenly on the grounded electrodes were treated for 10, 30, 60, 180 and 900 s.

Temperature during DBD treatments was monitored by means of a thermocouple welded to the grounded electrode and by using a pyrometer before and after the plasma treatments. A maximun increment of aproximately 1 °C in the grounded electrode was found after 15 min of operation (the dependence of temperature on operation time is reported as suplementary material in Figure S8 **)**. The low current through the plasma and the big area of the electrodes are reasons for this negligible temperature increase.

Chemical composition of plasma gas was analyzed by using an ozone sensor (Ozone Switch^TM^3, Eco Sensors Inc., United States) and an infrared spectrometer (Agilent Technologies Carry 630 FTIR, coupled directly to the DBD reactor) and operating the reactor under static conditions. Plasma chemistry was also analyzed by optical emission spectroscopy (Jobin-Yvon FHR640 - photomultiplier tube RP928, Hamamatsu Photonics K.K., Japan). Ozone was found to be the main product of the discharge, its concentration reached 10 ppm after half an hour of activation (see Figure S9 in the suplementary information). The recorded discharge emission spectra was characterized by the second nitrogen positive system, but neither NO^*^_x_ nor OH^*^ emissions could be detected (see supplementary information S4). This set of gas chemistry and emission spectroscopy results proves the oxidative character of the plasma and discards any substantial seed dehydration during plasma activation. On the other hand, although the UV photons (see Figure S4 in the supplementary information) coming from nitrogen plasma activated species (second positive system) are supposed to have little effect on germination due to their low intensity, similar wavelenght range than solar light UV photons and low penetration (some hundred of nanometers) within the seeds, some bond breaking events within the pericarp can not be discarded.

A parallel plate circular capacitive RF reactor of 10 cm electrodes diameter and 2.5 cm inter-electrode space, working at a pressure of 0.1 mbar (gas flow 5 cm^3^/min) was used for the low pressure plasma treatments. A RF (13.56 MHz) power of 15 W was applied to the bottom electrode, while the top electrode was grounded together with the rest of the plasma chamber. A self-induced negative bias voltage of 200 V was generated on the bottom electrode acting as sample holder where the seeds were evenly distributed. During this treatment, and for periods longer than five minutes, no temperature increase was detected with thermostrips (Bürklin GmbH & Co. KG, Germany) stuck on the bottom electrode. The fact that the bottom electrode possesses an air refrigeration system favored this stabilization of the working conditions. A picture of the reactor is shown in Fig. 7b. Further details about the experimental setup can be found in a previous publication^55^. Vacuum air plasma treatments were carried out for periods of 10, 30, 60 and 180 s.

### Germination and water uptake tests

Quinoa seeds (140) were placed in 10 Petri dishes (14 in each of 9 dishes and 14 in one additional dish as control) on wet filter paper with distilled water. All the Petri dishes were placed in a culture chamber at 23 ± 1 °C of temperature, 30 μEm^−2^s^−1^ of light intensity and 16 h photoperiod. The seeds were watered every two days with 5 ml of distilled water to maintain the moisture content. Number of germinated seeds was counted at 2, 5, 6, 8 and 12 days. A seed was considered as germinated when root and cotyledons emerged from soil.

Seed water uptake was firstly determined by monitoring the weight increase of 100 seeds soaked in tap water. At given times (0, 1.75, 3, 5, 24 and 48 h) the seeds were removed from the water using a strainer, surface dried with filter paper and weighted. Immediately afterwards the seeds were returned to the water until the next measurement.

Equivalent water uptake experiments were also carried out for the original and plasma treated seeds place in a close environment with saturated water vapor at room temperature. The weight increase after successive exposure experiments was taken as a measure of the water incorporated into the seeds as a function of time up to 1500 min. The conditions of this experiment were also used for seeds investigated by XPS after plasma treatment.

For all kind of experiments, statistical analysis was carried out using IBM SPSS Statistics v.22. Differences in percentages cases were compared using z test. The results are based on two-sided tests with a significance level of 0.05%. The tests are set for all pairwise comparisons using Bonferroni correction.

### XPS and SEM analysis

Quinoa seeds were analyzed by XPS in a PHOIBOS spectrometer using the Mg Kα as excitation source and working in the pass energy constant mode. Binding energy scale of the spectra was referred to the the main C1s signal peak attributed to C-C and C-H bonds taken at 284.5 eV. Spectra were recorded for untreated and plasma treated seeds for different times and for these latter seeds taken out from the spectrometer and exposed to air saturated with water vapor at room temperature for 24 h in a close container under the conditions described above for weight increase experiments. Note that prior to recording the spectra, samples had to be kept in the prechamber of the spectrometer up to reach the base pressure required to collect the spectrum.This conditioning time under ultrahigh vacuum conditions lasted for about 24 hours. For the XPS analysis a variable number of seeds were piled up to completely cover the porthole sample, thus avoiding any contribution from this latter to the recorded signals.

The surface morphology of the Quinoa seeds was also examined by means of a HITACHI 4800 scanning electron microscope working at 2 kV. EDX maps were adquired with a Bruker-X Flash-4010 working at 20 kV (aprox. depth analysis of 1 micron).

## Electronic supplementary material

Supplementary Info
