# Supplementary material for: Surface chemistry and germination improvement of Quinoa seeds subjected to plasma activation
Source: Sci Rep. 2017 Jul 19;7:5924. doi: 10.1038/s41598-017-06164-5 (PMC5517418; doi:10.1038/s41598-017-06164-5)
Supplement: Supplementary file 1 — Supplementary Info [file 41598_2017_6164_MOESM1_ESM.pdf]

Surface chemistry and germination improvement of Quinoa seeds subjected to plasma activation

A. Gómez-Ramírez[1,2‡*], C. López-Santos[2‡], M. Cantos[3], J. L. García[3], R. Molina[4], J. Cotrino[1,2], J. P. Espinos[2], A. R. González-Elipe[2*]

‡ These authors contributed equally to this work.

[1]*Departamento de Física Atómica, Molecular y Nuclear, Universidad de Sevilla, Avda. Reina Mercedes, 41012 Sevilla, Spain.*

[2]*Laboratory of Nanotechnology on Surfaces. Instituto de Ciencia de los Materiales de Sevilla (CSIC-Universidad de Sevilla), Avda. Américo Vespucio 49, 41092 Sevilla, Spain.*

[3]*Department of Plant Biotechnology, Instituto de Recursos Naturales y Agrobiología de Sevilla (CSIC), Av. Reina Mercedes, 10, Sevilla 41012, Spain*

[4]*Institute of Advanced Chemistry of Catalonia (IQAC), Department of Chemical and Surfactants Technology, Plasma Chemistry Group, Consejo Superior de Investigaciones Cientificas (CSIC), C/Jordi Girona 18-26, 08034 Barcelona, Spain.*

SUPPLEMENTARY INFORMATION

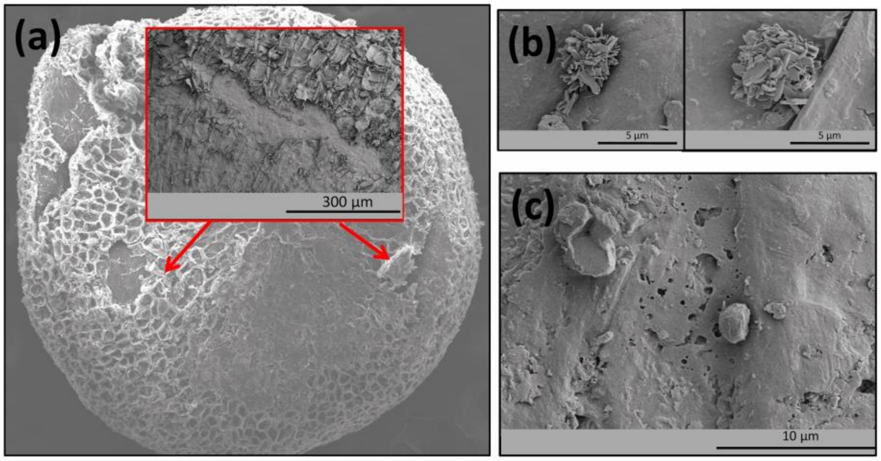

Figure S1. SEM micrographs of RF plasma treated Quinoa seeds (180 s). Parts (a) and (c) show the occurrence of surface damages on the pericarp, while (b) shows the formation of segregated agglomerates on the surface.

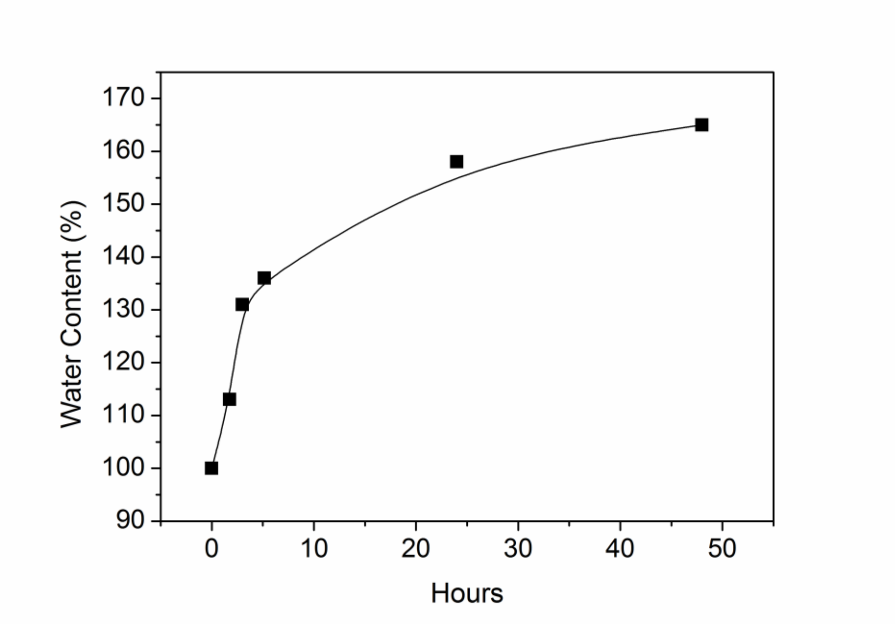

Figure S2. Percentage evolution of water uptake of original quinoa seeds for a period of 48 hours after first immersion in water.

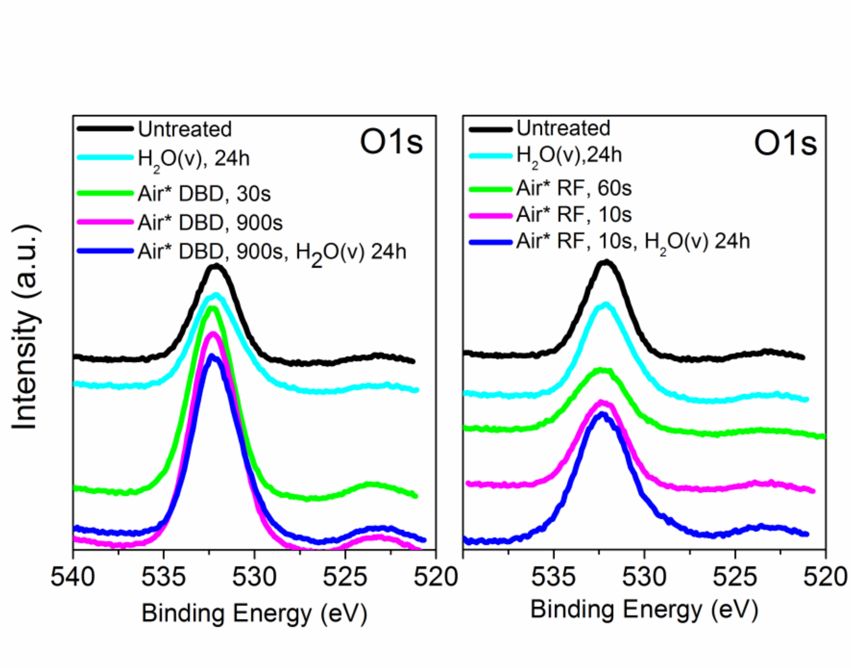

Figure S3. Oxygen O1s high-resolved XPS spectra for DBD and RF plasma treated Quinoa seeds at different treatment times in comparison with untreated seed spectra.

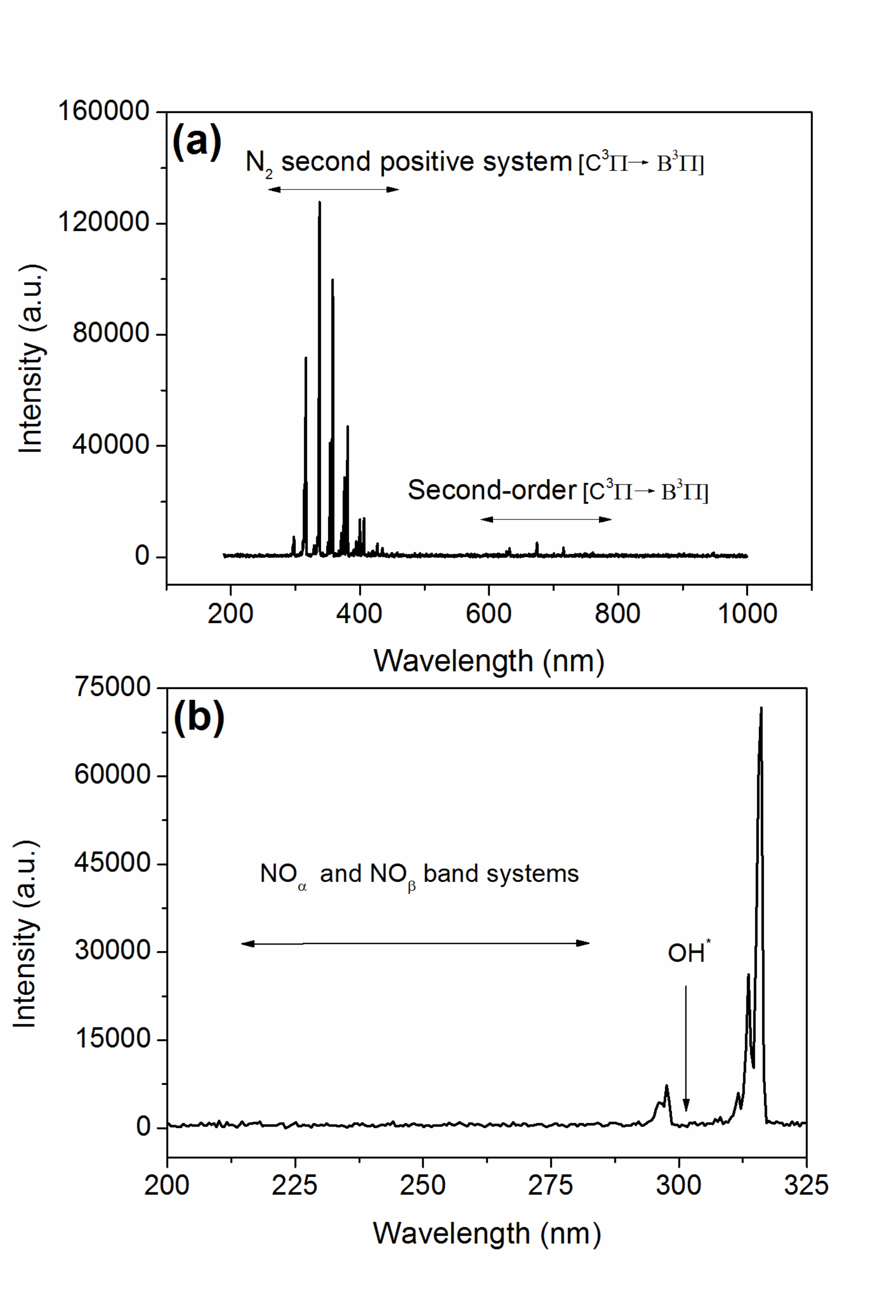

Figure S4. Optical emission spectra of the DBD discharge after 15 min of treatment. Part (a) shows the second positive system of nitrogen, while in part (b) we observe the absence of OH^* and NO_x species in the gas composition (entrance and exit slit apertures 0.5 mm, diffraction grating blazed at 330 nm, integration time 0.01 s and resolution 0.05nm, photomultiplier tube spectral sensitivity 25-60% in the range 200-300 nm).

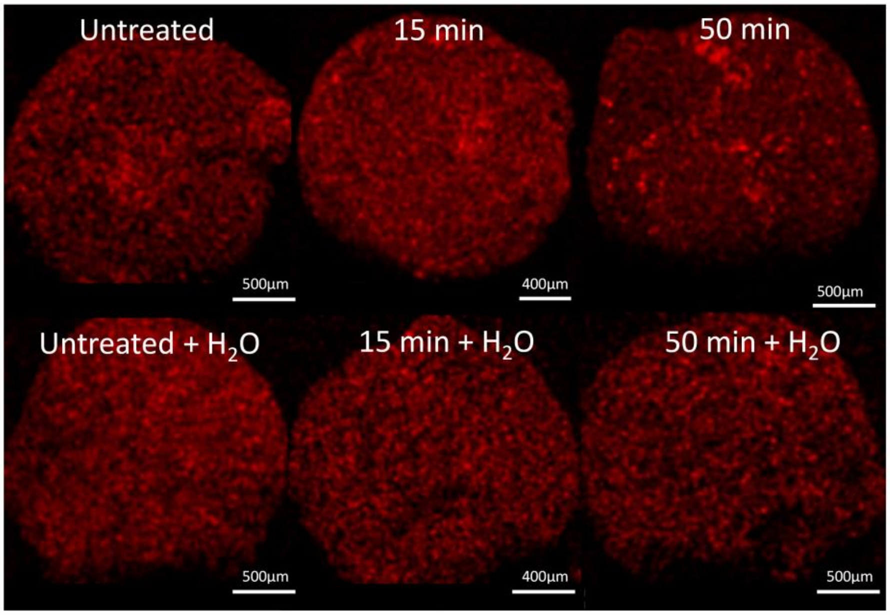

Figure S5. Top) EDX maps of potassium for the original and DBD plasma treated seeds for the indicated periods of time. Bottom) The corresponding K maps after 24 h exposure to water vapour.

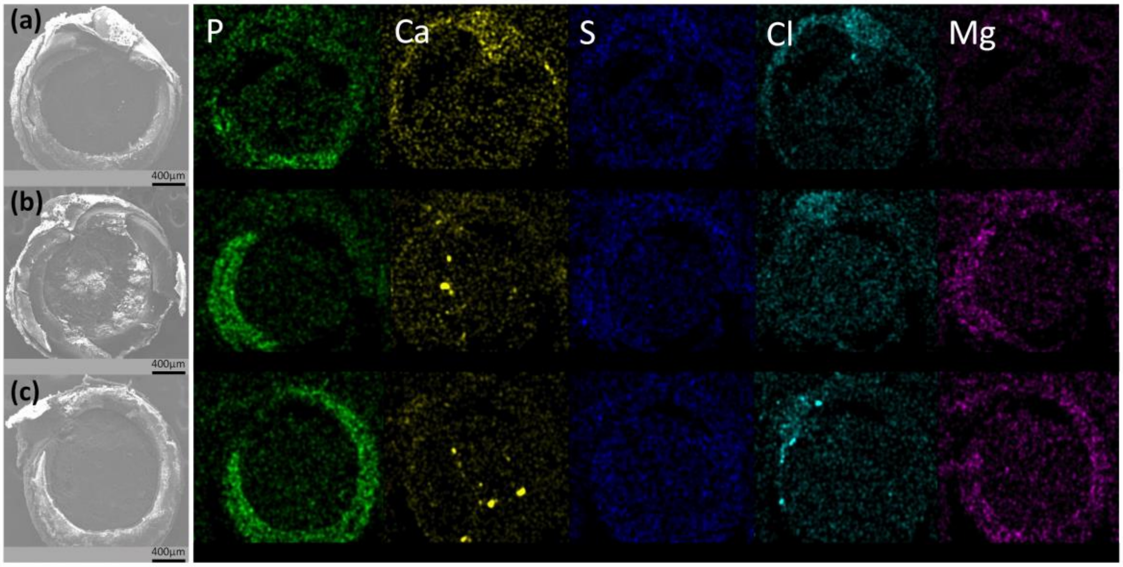

Figure S6. Left) Low magnification SEM micrographs of Quinoa seed transversal sections after DBD plasma treatments for different periods of time: (a) 0 s, (b) 15 min and (c) 50 min. Right) the corresponding EDX maps of P, Ca, S, Cl and Mg.

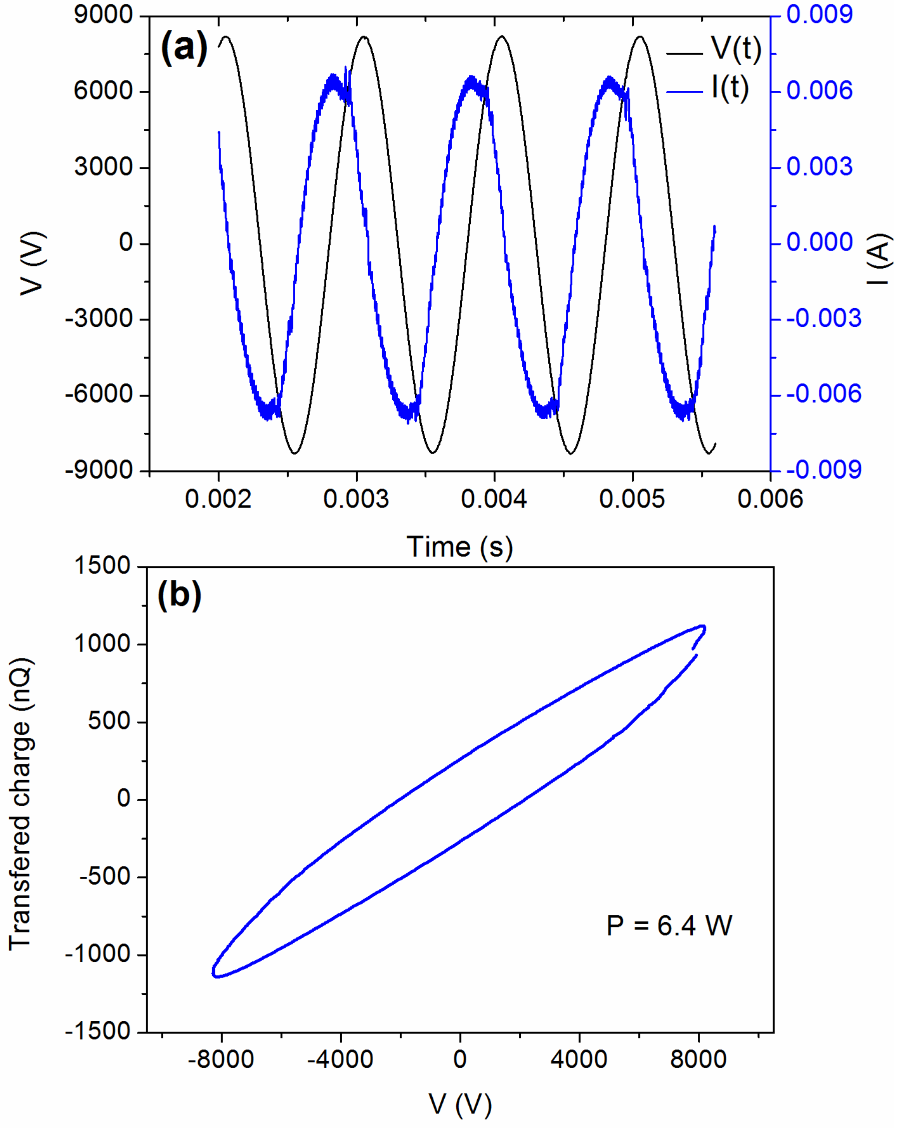

Figure S7. (a) I(t) and V(t) curves and (b) Lissajous plot for the DBD reactor operated at 1 kHz and 8.2 kV during seed treatments.

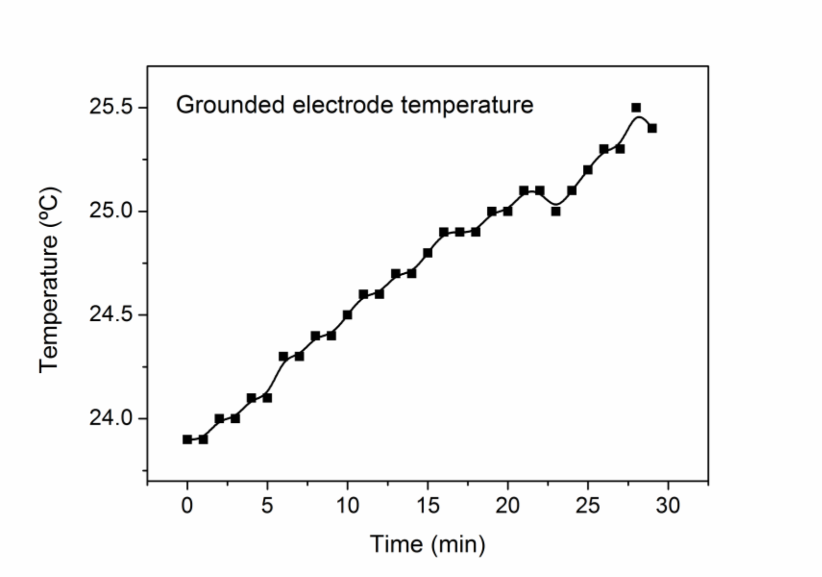

Figure S8. Grounded electrode temperature evolution during the DBD seed activation measured by means of a thermocouple welded to the grounded electrode. Using a pyrometer focused on the grounded or active electrode the temperature increase was 1ºC and 2.5 ºC, respectively.

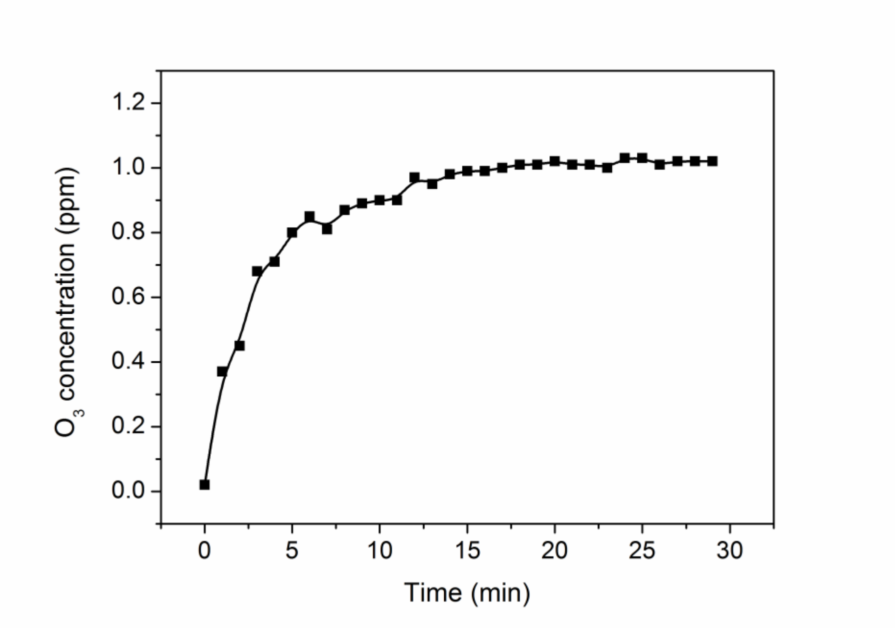

Figure S9. Evolution of ozone concentration during DBD seed plasma activation treatments.
